# RASOnD - A comprehensive resource and search tool for RAS superfamily oncogenes from various species

**DOI:** 10.1186/1471-2164-12-341

**Published:** 2011-07-05

**Authors:** Umay Kulsum, Vishwadeep Singh, Sujata Sharma, A Srinivasan, Tej P Singh, Punit Kaur

**Affiliations:** 1 Department of Biophysics, All India Institute of Medical Sciences, New Delhi-110029, India

## Abstract

**Background:**

The Ras superfamily plays an important role in the control of cell signalling and division. Mutations in the Ras genes convert them into active oncogenes. The Ras oncogenes form a major thrust of global cancer research as they are involved in the development and progression of tumors. This has resulted in the exponential growth of data on Ras superfamily across different public databases and in literature. However, no dedicated public resource is currently available for data mining and analysis on this family. The present database was developed to facilitate straightforward accession, retrieval and analysis of information available on Ras oncogenes from one particular site.

**Description:**

We have developed the RAS Oncogene Database (RASOnD) as a comprehensive knowledgebase that provides integrated and curated information on a single platform for oncogenes of Ras superfamily. RASOnD encompasses exhaustive genomics and proteomics data existing across diverse publicly accessible databases. This resource presently includes overall 199,046 entries from 101 different species. It provides a search tool to generate information about their nucleotide and amino acid sequences, single nucleotide polymorphisms, chromosome positions, orthologies, motifs, structures, related pathways and associated diseases. We have implemented a number of user-friendly search interfaces and sequence analysis tools. At present the user can (i) browse the data (ii) search any field through a simple or advance search interface and (iii) perform a BLAST search and subsequently CLUSTALW multiple sequence alignment by selecting sequences of Ras oncogenes. The Generic gene browser, GBrowse, JMOL for structural visualization and TREEVIEW for phylograms have been integrated for clear perception of retrieved data. External links to related databases have been included in RASOnD.

**Conclusions:**

This database is a resource and search tool dedicated to Ras oncogenes. It has utility to cancer biologists and cell molecular biologists as it is a ready source for research, identification and elucidation of the role of these oncogenes. The data generated can be used for understanding the relationship between the Ras oncogenes and their association with cancer. The database updated monthly is freely accessible online at http://202.141.47.181/rasond/ and http://www.aiims.edu/RAS.html.

## Background

The driving force behind oncogenesis is the transformation of normal cells to uncontrolled cell proliferation and invasion. A number of genes involved in regulating the expression, growth and survival of cells have been identified in events leading to malignant transformation. Ras (RAt Sarcoma) is a major multigene superfamily that has been implicated in approximately 30% of the known human tumors [1]. The predominant cancers involving Ras are associated with lung [2], colorectal region [3,4], pancreas [5] and thyroid [6]. The activation of Ras from a proto-oncogene into an oncogene results from a point mutation in the gene [7]. The Ras oncogenes cause hyperactive cell signalling and consequently contribute to the abnormal growth of the cell. These oncogenes have also been identified in human developmental disorders [8]. The Ras genes are expressed in nearly all tissues, though their expression levels may differ extensively. The most frequently observed Ras genes in human tumors are HRAS (Harvey-Ras), KRAS (Kristen-Ras) and NRAS (neuroblastoma-Ras) which vary in nature and specificity according to the cancer type [9].

The Ras proto-oncogene encodes a 21 kDa (p21) small monomeric guanine nucleotide-binding protein. The Ras proteins play a central role in the control of normal and transformed cell proliferation. They have the ability to bind both guanosine triphoshate (GTP) and guanosine diphosphate (GDP) and function as a molecular switch in signal transduction by alternating between the inactive GDP-bound state and the active GTP-bound form. The Ras in the active form signals cell growth whereas in the inactive state it cannot initiate these pathways. This binary switch system of the Ras protein is localized to conformational changes in two distinct regions comprising switch I and switch II [10]. The structural changes in the mutated Ras hinder its ability to hydrolyse GTP. The molecular switch gets trapped in the 'switch on' state resulting in increased Ras-GTP levels. The signalling pathway is thus continuously stimulated which leads to oncogenesis [11]. The most commonly observed point mutations in human tumors are at codons 12/13, or 61. Mutations involving residues 59, 63, 116, 117 and 119 have also been implicated in the oncogenic activation by Ras protein [12].

Ras was the first oncogene to be discovered among the transforming genes of the Harvey and Kirsten murine sarcoma viruses [13,14]. Since then a large number of Ras genes and proteins have been identified from different species including humans. On the basis of sequence similarity to the founding members [15], the Ras superfamily has been broadly classified into five main families, Ras [16], Rho [17], Arf [18], Rab [19] and Ran [20]. An additional family 'Others' has been assigned where their function is not clear. The Ras superfamily has grown and presently consists of over 150 members from humans [21]. Its orthologs have also been identified from nearly 100 other species. This superfamily has been the subject of several general [12,21-24] and specific reviews emphasizing their role in human and experimental cancer [25], cell signaling and transformation [26,27], cell motility [28], and differential functions in different tumors [29]. Most researchers across the globe have focused mainly on the identification, activation and clinical significance of these oncoproteins. This has led to the accumulation of enormous amount of data across numerous databases and in literature. There is no single database to our knowledge, presently available, where all information is contained on a single platform. This knowledgebase, compiles data from sequence to functional level accessible across diverse databases to enable the user rapid access, retrieval and analysis of information from one location. Thus, RASOnD aims to provide a better understanding of the Ras genes and proteins, their relationship with respect to each other and cancer.

## Construction and Content

### Data Generation

The database was developed with the objective to allow simple retrieval and exploratory analysis of information related to Ras oncogenes at a single point. It harbors a total of 199,046 entries from 101 species (Table 1). The details in RASOnD have been extracted from diverse public primary databases. The databases from which the information has been derived include the various resources available at National Centre for Biotechnology Information (NCBI) like GenBank [30] and Online Mendelian Inheritance in Man (OMIM), Kyoto Encyclopedia of Genes and Genomes (KEGG) [31], Database of Protein domains, families and functional sites (PROSITE) [32], Universal Protein Resource (UniProt) [33], Protein Databank (PDB) [34] and literature (PubMed). Various different keywords related to 'Ras' were used for extraction of data. The keywords combined with Ras included 'gene/oncogene', mutation, disease, expression, eukaryote and terms related to cancer like 'carcinoma/carcinogenesis/oncogenesis/tumor/hyperplasia/malignancy'. The search criteria also included expressions related to Ras like 'small monomeric G-proteins', 'GTP-binding proteins', 'small GTPases' and 'Ras related GTPases'. We have also annotated the data according to the associated cancer type as available in KEGG, OMIM and PubMed before April, 2011. Links to external databases have been included within each entry.

**Table 1:** Overview of the Information content in RASOnD

Data Type	Number
Species	101

Ras Families (excluding sub-families)	6

Nucleotide FASTA Sequences	4,882

Protein FASTA Sequences	4,882

Single Nucleotide Polymorphisms (SNPs)	102,426

Chromosome Positions	1,033

Pfam Entries	16,161

Prosite Entries	1,656

Protein Profiles	61,025

Protein Patterns	4,900

PDB Structures	548

Kegg Orthologies	152

Kegg Entries	3,871

Related Diseases	30

Related Pathways	3,626

### Implementation Detail

**Figure 1:**
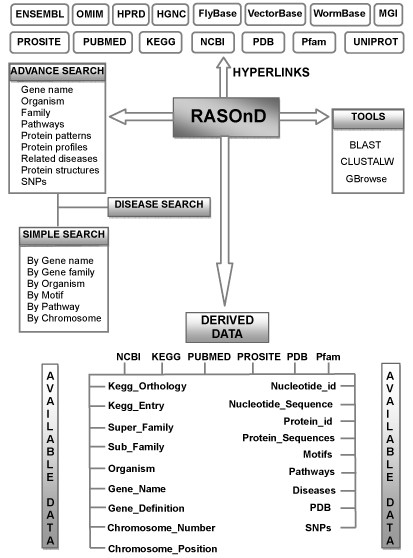
**RASOnD Database scheme**. The conceptual scheme depicting the various components of the database.

We have developed and implemented RASOnD using freely available online open source softwares. Apache HTTP Server serves as the localhost with MySQL relational database management system at the backend for storing and maintaining the data. The front end is designed with the help of HTML and CSS whereas the dynamic pages have been created through PHP. Parsing scripts written in PERL using regular expression were used to download data. The data is divided into 15 core tables with details on gene description, their family and sub-family, FASTA sequences - both nucleotide and protein, patterns, profiles, chromosome positions, related pathways, single nucleotide polymorphisms (SNPs), structures and links to related databases (Figure 1). The generic genome browser, GBrowse [35], open source molecular viewer JMOL [36] and TreeView [37] for phylograms have been implemented for effortless visualization and analysis. The user queries retrieve information from relational database tables which is displayed on the web interface. A local BLAST [38,39] and CLUSTALW [40] tool can be used to retrieve and compare sequences of interest from within the database.

### Utility

#### User Interface - Simple and Advanced Search

The interfaces in RASOnD are designed to facilitate straightforward navigation and exploitation of tools integrated in the database. The data stored in the RASOnD can be accessed employing a variety of search queries which allow the user to retrieve and analyze the data in a simple way. The users can retrieve information from the database through two user friendly interfaces comprising a 'Simple' and an 'Advanced' search. A search through the database can be performed separately on a particular feature in the simple search as well as simultaneously on multiple fields using the advanced search option. The simple search allows the user to explore the database by selecting a specific individual field from the drop down menu. The user can perform a search by entering the keyword or by selecting the options available in the interface. Various alternatives are present in the simple search.

**Figure 2:**
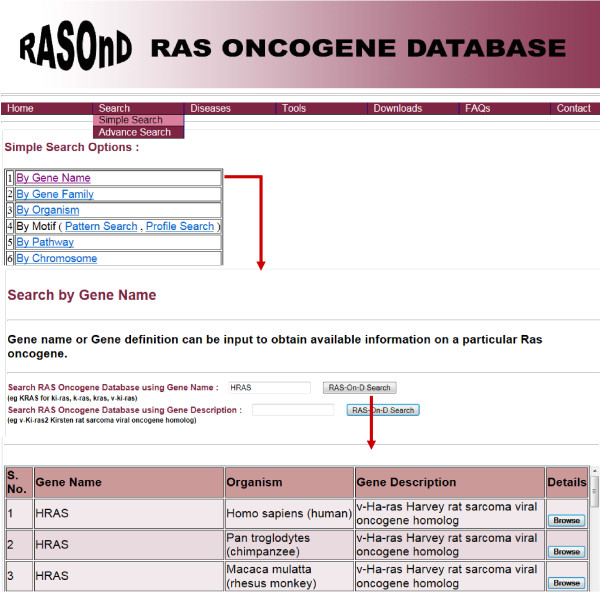
**Screen shots of User Interface - Simple Search**. Simple search query using one of the six options provided e.g. Gene Name. This query on providing the gene name results in a page containing the organism name, the gene description and a 'Browse' button. A click on this button provides details related to that particular gene. The red arrows indicate the pages obtained on clicking the 'Submit' button

The "gene name or INSD (International Nucleotide Sequence Database) accession number" can be input by the user to extract all available information on a particular Ras oncogene (Figure 2). The "gene family" option allows the investigator to select a family from the five main families of Ras superfamily. The option of further selecting a member from the subfamilies of Ras, Rab, Rho, Ran, Arf/Sar family and Others is also incorporated. The information on all Ras oncogenes present in a specific species can be retrieved by choosing the "organism search" option from the dropdown menu. The "motif search" allows the user to scan motifs related to a gene. This comprises a pattern and a profile search which generates data on exisiting patterns and predicted motifs respectively obtained from PROSITE scan. The user can also opt for the pathway of interest where these oncogenes are involved via the "pathway search" menu. The "chromosome position" alternative permits the user to access the details for genes present in a desired organism. Selection of the desired organism will present a table pertaining to the chromosome number and position and related KEGG and NCBI entry.

**Figure 3:**
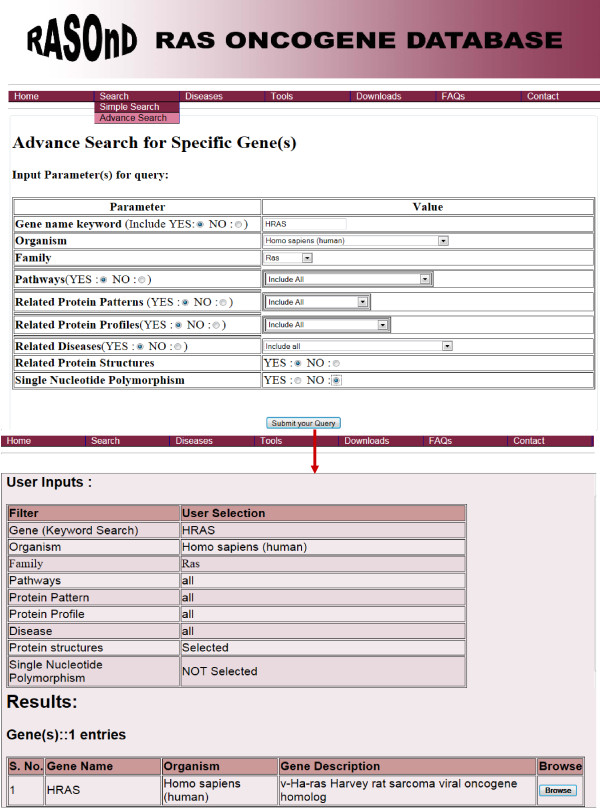
**Screen shots of User Interface - Advanced Search**. The advanced search interface where more than one option can be selected simultaneously to obtain varied information about the Ras gene/protein. The user can choose to include or exclude any of the options provided. The user has to input the gene name of interest and then further pick either a parameter from the drop down menu or decide on one of the default buttons incorporated for the search. Submission of this option returns the genes according to the search criteria. A click on the 'Browse' button will return detailed data on the selected gene.

We have incorporated the 'Advance search" option to allow search of multiple fields simultaneously. This search includes all the fields available in the simple search menu. The user in this interface can select specific features and thus restrict the field of search. The user can (i) input the gene or explore all genes based on the subsequent parameters (ii) use the different aspects incorporated as a drop down menu to access all or one entry from the various parameters like organism, Ras family, related pathways, patterns, profiles and disease and (iii) decide to include or exclude the protein structures and SNPs. Thus, the user can narrow down the search to the requisite criterion by utilizing this interface (Figure 3). The initial output from this interface displays the user selection before providing the tabulated results.

#### User Interface - Diseases

The Ras oncogenes have been implicated in a variety of cancers and developmental disorders. The human cancers which have the presence of Ras oncogenes are included as a dropdown menu under the 'Disease' section. The user can choose the disease of interest from this menu. On selection, initially a summary table on human Ras members involved in the preferred disease is returned with a 'Browse' button. The related Ras oncogenes/proteins from other species are also indicated. This page also furnishes a short description of the cancer type.

#### User Interface - Results

**Figure 4:**
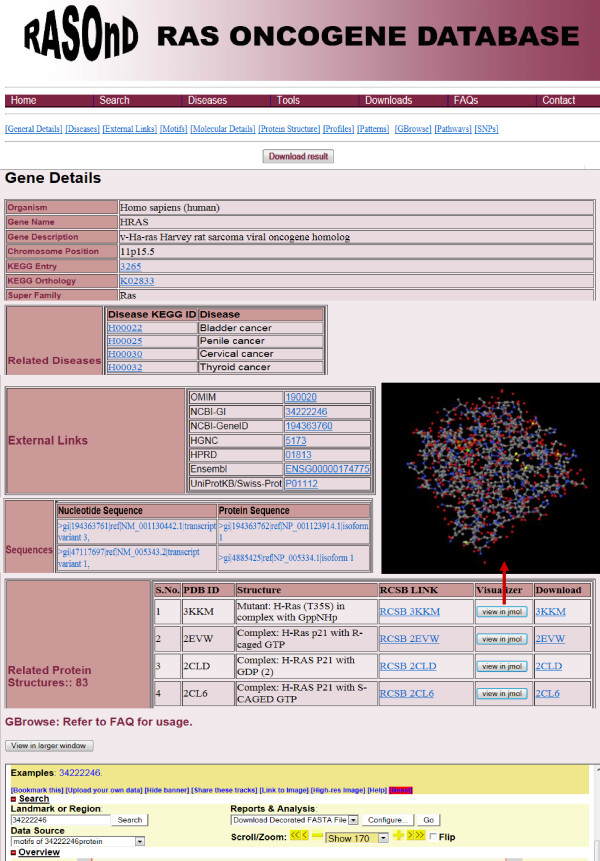
**Screen shots of User Interface - Result Page**. Collage of some of the results produced via the 'Browse' button in both 'Simple' and 'Advanced' search options.

The results for each search are displayed in two parts. An initial investigation using any of the search options returns a summary table displayed in tabular format listing the Ras oncogenes from different species with a 'Browse' button. A click on this button presents additional and detailed records on varied aspects of the selected oncogene like gene name with the nucleotide and protein sequences, KEGG orthology, pathways, chromosome position, available structures, motifs and SNPs for all the genes belonging to the selected organism (Figure 4). The diseases with Ras manifestation are also exhibited which have been further hyperlinked to KEGG. Users can graphically view the motifs via the GBrowse tool. The PDB Ids have been reported where the three-dimensional structure is available. The coordinates can be downloaded or structures analysed with the JMOL applet embedded in the database. A hyperlink to RCSB Protein Data Bank has also been integrated. Similarly, the nucleotide or protein sequence data at each point can be retrieved or a local BLAST run to obtain sequences similar to the one of interest. The homologous sequences returned from the BLAST search can be further selected for onward submission to CLUSTALW for multiple sequence alignment. The selection can be made via a click on the buttons provided on the edge of the sequence. We have also hyperlinked each entry to the related databases like HPRD, KEGG, OMIM and HGNC. In the Pathways page the results are presented as KEGG Pathway ID, related organism, KEGG Entry and pathway image which can be further chosen for obtaining detailed information on the oncogene. Links to the KEGG database are included for scrutiny of proteins implicated in that pathway.

#### WebTools

The analysis of the data within the database can be carried out using the tools available in the database. Three tools have been implemented. The users can utilize the genome viewer, 'GBrowse' for browsing associated genes and patterns and profiles of predicted motifs for the selected oncogene. The user can carry out sequence similarity searches from within the database using 'RAS-ON-DBLAST'. The user-defined query nucleotide or protein sequence in FASTA format can be submitted for a local BLAST search against the database to identify homologous sequences. The default or user-defined parameters like e-value cutoff, gap opening, word size or matrix can be modified for such a search. The user can choose sequences returned for direct onward submission to CLUSTALW or download the selected sequences for any other analysis. "CLUSTALW" can be used for the multiple sequence alignment of the selected sequences obtained from the database query. The user can also enter additional sequences of interest. The phylograms generated from CLUSTALW tool can be observed with TreeView.

#### Additional features

The home page includes a FAQ page with the purpose of providing basic facts on the Ras superfamily as well as the usage of the entire database. This page also includes a section which is hyperlinked to reviews available in literature. A download option has been provided to obtain data related to nucleotide sequences, protein sequences, single nucleotide polymorphisms and protein structures as separate files. Links to other databases which refer to some Ras superfamily members and latest available literature from PubMed have also been included on the home page.

## Discussion

The main focus of research in molecular cancer is not only the identification of the genes altered in different tumor types but also determination of the pathological role played by them in tumorogenesis. The Ras genes and proteins are particularly very significant as they are activated by point mutations and are the predominant oncogenes in human tumors. The comprehension of different single amino acid substitutions which lead to malignancy is increasingly becoming an important tool to elucidate the mechanisms of oncogenesis. Moreover, the Ras dependent pathways are now being targeted for the development of anti-cancer agents. The objective of this database is to amalgamate information distributed across diverse platforms to a single site. It stores data related to Ras genes and proteins, related polymorphisms, their pathways, associated diseases, motifs and structures. It also contains tools for the examination and analysis of these sequences. It thus, serves as an organized source to both clinicians and basic scientific community for research on Ras superfamily. This resource has been designed to allow the user to explore and extract effortlessly all accessible facts at one common place. RASOnD attempts to bridge the gap between the genomics and system biology and affords inputs and links to all aspects of Ras oncogenes not accessible as a central resource so far. RASOnD is of interest to molecular oncologists and researchers working in the field of cancer as it brings under one roof, data for which otherwise a search would have to be conducted across varied resources. It, therefore, serves as a useful platform for ready reference for identification, determination and comparative analysis of the genes and proteins belonging to the Ras superfamily.

### Comparison to other related Databases

There is no similar database to RASOnD, however, there exist a small number of databases which have some reference to the 'Ras' gene/protein "see Additional file 1, Figure S1". These deal with only a few species (unlike the present which includes information on all available species) or present specific rather than generalized and detailed information on the 'Ras' superfamily. Majority have a focus on genomics rather than a proteomics approach unlike RASOnD which provides detailed information on both aspects. These databases include ACTuDB [41], Catalog of Somatic Mutations in Cancer (COSMIC) database [42], Dragon database for exploration of ovarian cancer genes (DDOC) [43], kinase pathway database [44], mouse genome database [45], Oncogenomic Database of Hepatocellular carcinoma (OncoDB.HCC) [46], rat genome database (RGD) [47], Signal Transduction Classification (STCDB) [48], Tumor gene family of databases (TGDB) [49] and Genecards [50]. The rat and mouse genome databases are useful if the focus of search is on these species. COSMIC database contains information on published somatic mutations only in various cancers and refers to just four families of Ras in humans. The DDOC and OncoDB.HCC resources are specific to Ras members implicated in ovarian cancer and hepatocellular carcinoma respectively. The DDOC deals with human Ras members whereas OncoDB.HCC comprises details on rat and mouse besides human members. The ACTuDB contains information on the genomic profiling of tumors only with little emphasis on Ras oncogenes while STCDB has reference to a handful of Ras genes/proteins implicated in signal transduction. The Kinase pathway database refers to only seven species. The GeneCards contains the maximum number of Ras members after RASOnD, however, it also refers to only some species. A link to these databases is available on the home page.

RASOnD is distinct from these published on-line public databases. The main differentiating feature is the inclusion of genomics and proteomics data from all species where Ras members have been identified. Thus, it is the single, specialized largest repository of Ras oncogenes. The purpose of developing RASOnD was to provide a simple solution to clinicians and oncologists to extract information on this gene. Moreover, it affords the alternative of selecting the disease of interest rather than just the Ras oncogene. The initial exploratory sequence analysis from BLAST can further be exploited for multiple sequence alignment with CLUSTALW by a one step selection of the returned sequences. This is a feature unique to the present database. In this manner, the concept of RASOnD is different from other available databases.

### Future Directions

The present database focuses mainly on the genes and proteins included in the Ras superfamily, their integration and involvement in different tumors and provides tools for their analysis. We further plan to extend the database with a greater emphasis on the Ras proteins to include their post-translation modifications, interacting partners and inhibitors. Proteins containing the RAS-binding domain will also be incorporated into the database.

## Conclusion

The RASOnD knowledgebase is the first attempt to construct an easily accessible and handy platform on the multifaceted large Ras superfamily. RASOnD presents the end-user with all possible details for analysis and retrieval on various aspects of RAS oncogenes in a simple and user friendly manner. Details about various other databases with reference to the Ras have been included. The information in the database can be accessed and investigated by a single query search or by a combination of various queries. Alternatively, the researcher can determine the Ras oncogene involved in a particular cancer type by exercising the dropdown menu under diseases. JMOL, GBrowse and TreeView have been implemented for easy visualization whereas BLAST and CLUSTALW modules have been incorporated for comparative analysis of sequences within the database. The wealth of information available in RASOnD can thus be exploited by both bench side workers and bioinformaticians to carry out a comprehensive analysis on the Ras superfamily. The computational biologists can utilize the compiled data to develop computational prediction tools for novel mutations.

## Availability and Requirements

The RASonD database will be continuously updated and upgraded and is freely available at http://www.aiims.edu/RAS.html and http://202.141.47.181/rasond/ to academic and non-academic users. Java enabled client web browser is required for the usage of the tools GBrowse, Jmol applet and Treeview.

## Competing interests

The authors declare that they have no competing interests.

## Authors' contributions

UK and VS collected and compiled the database from public databases and literature and developed the database. AS refined the manuscript. SS and TPS coordinated the project. PK conceived and designed this study and prepared the manuscript. All authors have read and approved the final manuscript.

## Supplementary Material

Additional file 1 **Figure S1 - Comparison to other related databases**. A three-dimensional bar plot indicating the comparison of 'Ras - related' information incorporated in RASOnD with other related resources. Click here for file
