# Supplementary figures and images for: RASOnD - A comprehensive resource and search tool for RAS superfamily oncogenes from various species
Source: BMC Genomics. 2011 Jul 5;12:341. doi: 10.1186/1471-2164-12-341 (PMC3141677; doi:10.1186/1471-2164-12-341)

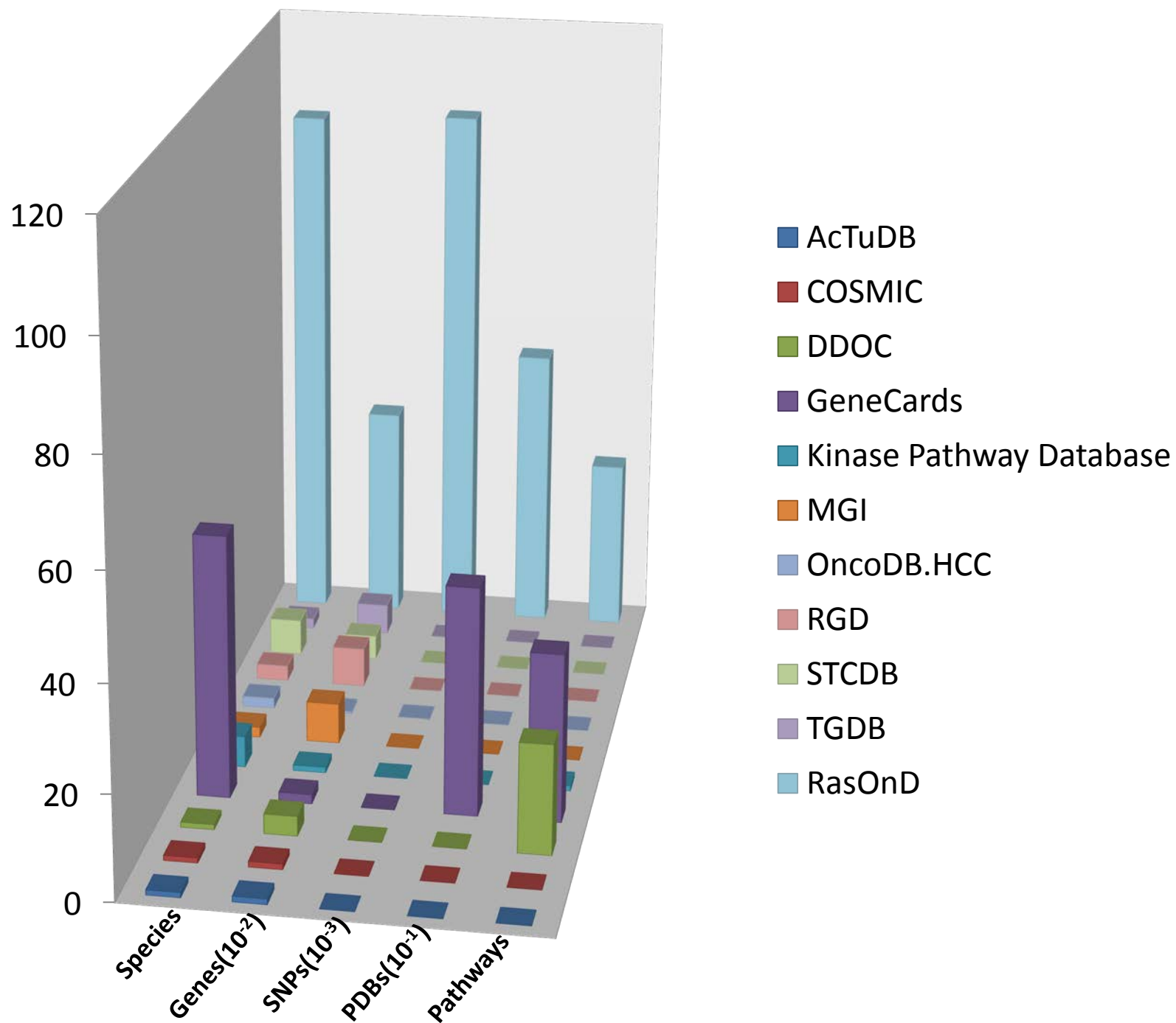

Supplement: Additional file 1 — Figure S1 - Comparison to other related databases. A three-dimensional bar plot indicating the comparison of 'Ras - related' information incorporated in RASOnD with other related resources. [file 1471-2164-12-341-S1.PDF]
